# Physicochemical characterisation of kafirins extracted from sorghum grain and dried distillers grain with solubles related to their biomaterial functionality

**DOI:** 10.1038/s41598-021-94718-z

**Published:** 2021-07-26

**Authors:** Umar Shah, Deepak Dwivedi, Mark Hackett, Hani Al-Salami, Ranjeet P. Utikar, Chris Blanchard, Adil Gani, Matthew R. Rowles, Stuart K. Johnson

**Affiliations:** 1 School of Molecular and Life Sciences, Faculty of Science and Engineering, Curtin University, GPO Box U1987, Perth, WA 6845 Australia; 2 Curtin Health Innovation Research Institute Curtin University, GPO Box U1987, Perth, WA 6845 Australia; 3 WA School of Mines, Mineral, Energy and Chemical Engineering, Curtin University, GPO BOX U1987, Perth, WA 6845 Australia; 4 Biotechnology and Drug Development Research Laboratory, School of Pharmacy and Biomedical Sciences, Curtin University, GPO BOX U1987, Perth, WA 6845 Australia; 5 ARC Industry Transformation Training Centre for Functional Grains, Charles Sturt University, Wagga Wagga, NSW Australia; 6 Department of Food Science and Technology, University of Kashmir, Srinagar, J&K 190006 India; 7 X-Ray Diffraction and Scattering, John de Later Centre, Curtin University, GPO BOX U1987, Perth, WA 6845 Australia

**Keywords:** Structural biology, Materials science

## Abstract

Kafirin, the hydrophobic prolamin storage protein in sorghum grain is enriched when the grain is used for bioethanol production to give dried distillers grain with solubles (DGGS) as a by-product. There is great interest in DDGS kafirin as a new source for biomaterials. There is however a lack of fundamental understanding of how the physicochemical properties of DDGS kafirin having been exposed to the high temperature conditions during ethanol production, compare to kafirin made directly from the grain. An understanding of these properties is required to catalyse the utilisation of DDGS kafirin for biomaterial applications. The aim of this study was to extract kafirin directly from sorghum grain and from DDGS derived from the same grain and, then perform a comparative investigation of the physicochemical properties of these kafirins in terms of: polypeptide profile by sodium-dodecyl sulphate polyacrylamide gel electrophoresis; secondary structure by Fourier transform infra-red spectroscopy and x-ray diffraction, self-assembly behaviour by small-angle x-ray scattering, surface morphology by scanning electron microscopy and surface chemical properties by energy dispersive x-ray spectroscopy. DDGS kafirin was found to have very similar polypeptide profile as grain kafirin but contained altered secondary structure with increased levels of β-sheets. The structure morphology showed surface fractals and surface elemental composition suggesting enhanced reactivity with possibility to endow interfacial wettability. These properties of DDGS kafirin may provide it with unique functionality and thus open up opportunities for it to be used as a novel food grade biomaterial.

## Introduction

The production of alcohol from grains such as maize and sorghum for use as biofuel is a current topic of commercial interest. Increased biofuel needs are predicted to occur over the next decade as reported by the EU Regulatory Framework for Biofuels^1^. Dried distillers grain with solubles (DDGS) is a protein enriched by-product from this industry, that remains after fermentation and distillation by heat treatment^2^. At present, some DDGS may be used as an animal feed supplement, but the rest is considered waste and may be dumped in sewers and rivers^3^. Unlocking value from unwanted DDGS is an important step to reduce this current environmental burden. Globally, several Authorities have identified priorities for the value-added utilisation of DDGS.

Kafirin is a hydrophobic storage protein found in sorghum grain. In the grain it contributes 65–75% of the total protein and it contains more than 50% hydrophobic amino acids. Efficient techniques for extraction and concentration of this major protein from sorghum grain have been reported along with its techno-functionality for use as a “green” polymer to replace synthetic ones^4^. Similarly the extraction of kafirin from sorghum DDGS has received some research attention but fundamental understanding of its physico-chemical properties related to biomaterial techno-functionality is still lacking^5,6^.

For potential application as a food grade biomaterial, kafirin satisfies all of the key characteristics namely: “GRAS” status, natural origin, biodegradable, low cost, non-allergic and abundant availability^7,8^. Kafirin from sorghum grain has gained interest because of its distinctive properties of: high hydrophobic to hydrophilic ratio, solvent induced self-assembling nature, high di-sulphide crosslinking, high gelling capacity, high stability and low digestibility^9^. The high hydrophobic to hydrophilic ratio is the principal characteristic that allows self-assembly of kafirin into various mesostructures such as spherical particles, films and fibres^4^. This hydrophobic nature is due to the large number of hydrophobic amino acid residues, for instance proline and amide nitrogen from glutamine (hence it belongs to the class of proteins called prolamins). This hydrophobicity along with exogenous (interactions of protein-non-protein) and endogenous factors (protein–protein interactions) gives kafirin its unique properties of resistance to hydration and slow digestibility^10^. Although, hydrophobic biomaterials are sought after, subtle modifications to kafirin such as increased surface hydrophilic sites might provide new opportunities for its use such as in designing biomaterials with unique targeted properties such as encapsulating agents for controlled release of bioactives in the gastrointestinal tract.

Kafirin shares a large degree of homology with zein (maize prolamin) with respect to their primary and secondary structures. Therefore published structure–function relationships of zein have been used as a model for understanding kafirin properties^10^. Only a few studies have been reported on industrial DDGS kafirin and/or heat-treated sorghum protein^11–14^. However, a complete structural and/or surface elemental analysis with regards to material functionality has not been studied yet, hindering the potential use of these proteins for development of new value-added biomaterials.

The aim of this study was to extract kafirin from sorghum grain and its DDGS, then to do a comparative investigation of their physico-chemical proteins in comparison with commercial zein in terms of: polypeptide profile by electrophoresis; secondary structure by Fourier transform infra-red spectroscopy (FTIR) and x-ray diffraction (XRD); self-assembly behaviours using small-angle x-ray scattering (SAXS); morphological imaging and surface chemical composition by scanning electron microscopy (SEM) and energy dispersive x-ray spectroscopy (EDS). These physico-chemical investigations will evaluate if the sorghum DDGS kafirin may have useful techno-functionality for future biomaterial applications.

## Material and methods

### Material

Ten kg each of whole grain sorghum and sorghum DDGS produced from the same whole grain were gifted by Dalby Bio-refinery, (Dalby, Queensland, Australia). The samples were vacuum packed and stored at 4 °C before further use.

Zein (grade: 99%; MW: 22–27 kDa), sodium hydroxide (NaOH; ACS regent grade: ≥ 98%; MW: 40 g/mol), sodium metabisulphite (Na_2_S_2_O_5_; ACS regent grade: ≥ 97.0%; MW: 190.11 g/mol), n-hexane (C_6_H_14_; ACS regent grade: 99%: MW: 86.18 g/mol), and methanol (CH_3_OH; ACS regent grade: 99.8%; MW: 32.04 g/mol) were obtained from Sigma Aldrich (Castle-Hill, NSW, Australia), absolute ethanol (CH_3_CH_2_OH; ACS regent grade: 95.5%; MW: 46.07 g/mol) and hydrochloric acid (HCl: ACS regent grade 37%; MW: 36.46 g/mol), were purchased from Thermo-Fisher Scientific (Scoresby, VIC, Australia). All the experiments were performed in Curtin University in accordance with the research related norms laid down by University following the Australian Code for the Responsible Conduct of Research.

### Extraction of kafirin

The extraction method was based on that of Lau et al.^2^ with minor modifications. The whole grain and the DDGS were milled into fine powder using a pin mill (Cemotec 1090 sample mill, Foss Tecator, Mulgrave, VIC, Australia) followed by blending (ZM 200 blender, Retcsh Gmbh & Co, Haan, Germany). The milled samples were passed through a 500 micron sieve with 85% recovery of the sieved fraction. Then the total sample was reconstituted.

The milled sorghum grain (duplicate × 50 g) and the DDGS (duplicate × 50 g) were soaked in 250 mL of extraction solution of 62% (v/v) absolute ethanol, 0.064 M NaOH and 0.22% (w/v) Na_2_S_2_O_5_ followed by 1 h of incubation at 60 °C with shaking at 150 rpm in a water bath (Memmert 854, Schwabach, Germany). The samples were then cooled to 25 °C followed by sonication in an ultrasound water bath (frequency 30 Hz, power 60 W; Ultrasonic cleaner, DSA, Madrid, Spain) for 5 min at 25 °C. The sonicated samples were then centrifuged (Eppendorf Centrifuge 5810 R, Macquarie Park, NSW, Australia) at 1750 *g* for 20 min at 4 °C. The clear supernatant that contained the dissolved kafirin was recovered by decantation. Vacuum rotary evaporation at 80 °C was used to reduce the total volume of the supernatant from 168 to 98 mL. Then 6 N HCl was used to adjust the pH of the concentrated supernatant to 5.0, resulting in precipitation of the kafirin. The samples were left overnight to complete the precipitation, followed by centrifugation at 1750 *g* for 10 min at room temperature. The supernatant was removed by decantation and the kafirin precipitate was dried overnight at 40 °C in an oven (Memmert, 854 Schwabach, Germany) to give a dried kafirin pellet.

The dried kafirin was defatted three times by washing with absolute n-hexane (40 mL/12 g) by initial manual shaking followed by standing at room temperature for 5 h. The solvent was removed by decantation, the residual solvent in the defatted kafirin was evaporated by heating for 24 h at 60 °C in an oven (Memmert 854\, Schwabach, Germany).

The purified kafirin was blended at ~ 500 *g* for 5 min (ZM 200 blender, Retcsh Gmbh & Co, Haan, Germany) to reduce and obtain a more uniform particle size. Particle size of the kafirins was determined using a using Master Sizer 2000 (Malvern Instruments Ltd, Malvern, UK) based on the Mie and Frunhofer scattering technique^15^. The particle size of grain kafirin and DDGS kafirin were 284 µm and 272 µm respectively.

The protein content of the kafirins was determined in triplicate by elemental analysis (2400, Perkin Elmer Pvt Ltd, Macquarie Park, NSW, Australia) which measured the nitrogen content. A conversion factor of 6.25 was used to convert percentage nitrogen to protein. The DDGS kafirin and the grain kafirin had 84.76 ± 0.76 g/100 g dry basis and 78.12 ± 0.51 g/100 g dry basis protein respectively (mean ± SEM; n = 3). The extraction yield of ~ 59 and ~ 38% was achieved for DDGS kafirin and grain kafirin respectively.

### Electrophoretic profile of proteins

Sodium dodecyl sulphate–polyacrylamide gel electrophoresis (SDS-PAGE) under non-reducing conditions was used to examine the protein profile of DDGS kafirin, grain kafirin and zein. A 4% stacking gel and 12% separating gel (In-vitrogen, Life Technologies Corp, Sydney, Australia) was used with tris–HCl/glycine running buffer (1.5 M) in a Mini-Protean II system (X Cell Surelock™ Mini-cell, electrophoresis unit (In-vitrogen, Carlsbad, California, USA). Five milligrams of DDGS kafirin, grain kafirin and zein were dissolved in 1.5 ml of non-reducing sample buffer (0.01 mL/mL Tris–HCl buffer (pH 6.8), 10% (v/v) SDS and 20% (v/v) glycerol). The In-vitrogen mark 12™ unstained standard was used as standard protein marker (In-vitrogen, Life Technologies Corp, Sydney, Australia).

The samples were heated in a boiling water bath with stirring for 25 min to completely dissolve the proteins. The prepared solutions (10 μl) were loaded onto the gel and electrophoresis was run at 200 V for 30 min until the leading edge of the migration was close to the bottom of the gel. The gels were stained for 20 h using 0.1% Bio-safe Coomassie G250 stain (Bio-Rad Laboratories, Carlsbad, California, USA). The stained gels were de-stained using methanol/acetic acid/water at 1:1:8 (v/v/v). The de-stained gels were imaged using a Bio-Rad Universal Hood II gel imaging system (Bio-Rad Laboratories, Hercules, CA, USA).

The migration of the sample bands was compared with that of the molecular weight standard mixture to estimate their molecular weight and identify their subunits based on molecular weights reported in the literature^2^.

### Attenuated total reflection-Fourier transform IR spectroscopy (ATR-FTIR)

The secondary structure of DDGS kafirin and grain kafirin was investigated on five replicates with attenuated total reflectance Fourier transform infrared spectroscopy (ATR-FTIR). ATR-FTIR spectra were collected using a PerkinElmer TWO ATR-FTIR spectrophotometer (PerkinElmer, Boston, Massachusetts, USA). This instrument is coupled with a single-bounce diamond ATR crystal. Spectra were recorded across the range of wavenumbers 4000–450 cm^−1^ at 4 cm^−1^ spectral resolution, with 128 co-added scans. Background spectra were recorded from the clean crystal to reduced beam current decay. Recorded spectra were analysed using OPUS software (V 7.0, Bruker, Elltingen, Germany). Specifically, spectra were vector normalised to the amide I band (1590–1710 cm^−1^) and the background corrected using the rubber correction method^16^. Prior to curve fitting, second-derivative spectra were calculated using a 13 smoothing point Savitzky-Golay smoothing function, with the number and position of the second-derivative minima, across the amide I range (1590–1710 cm^−1^) used for curve fitting (specifically, 9 bands centred at 1686, 1677, 1669, 1660, 1650, 1643, 1634, 1623, and 1610 cm^−1^). The area under examined multiple overlapping absorbance bands of amide I was used as implication standard for estimating relative changes in position and shape of secondary structure of examined proteins.

### X-ray diffraction (XRD) analysis

X-ray diffraction was applied to help reveal differences in phase and crystallite size between the protein samples. In brief, this analysis was carried out in duplicate using a D8 Advance powder diffractometer (Bruker AXS, Karlsruhe, Germany) with a Cu Kα (*λ* = 1.54 Å) radiation source (@40 kV & 40 mA) with a LynxEye detector. The diffraction patterns were collected over the range of 7.5–90° 2θ with the step size of 0.015° 2θ for 60 min.

Crystallite size is known to affect various key properties such as solubility, stability, and molecular interactions. The crystallite size was calculated using the double-Voigt method^17^ where the peaks are described by a pseudo-Voigt peak and the final crystallite size extracted from its integral breadth. The fittings of the data was performed by full pattern data analysis programme, TOPAS (Bruker, Karlsruhe, Germany).

### Small-angle x-ray scattering (SAXS)

Particle size in solution of DDGS kafirin, grain kafirin and zein were examined using SAXS. Data were collected using a Bruker MetalJet Nanostar (Bruker AXS, Billerica, Massachusetts, United States) over a *q* range of 0.008–0.35 Å^−1^. In brief, 10 mg/mL solutions of sample in 62% aqueous ethanol and loaded into a capillary tube sample holder. Background patterns were collected for 300 s, followed by exposing the samples with X-rays (1.34 Å) for 3600 s. Background subtraction was performed for each spectrum. The Irena software package^18^ was used for data modelling using the unified-fit approach^19^.

### Field emission-scanning electron microscopy (FE-SEM) and energy-dispersive X-ray spectroscopy (EDS)

The surface morphology of DDGS kafirin, grain kafirin and zein was investigated using secondary electron (SE) imaging on a dual-beam field emission scanning electron microscope (Zeiss Neon 40EsB FEBSEM, Oberkochen, Germany). Samples were kept in a desiccator then placed onto aluminium stubs using carbon tape and coated with 6 nm of platinum using a splutter coater (208HR, Cressington, Watford, UK). A 5 kV electron beam was used^20^. Surface elemental composition was determined using EDS at higher kV tailored with the FEB-SEM^21^. Surface elemental distribution was collected over various areas and elemental identification was performed by Aztec software. (Oxford Instruments, Wiesbaden, Germany).

## Results and discussion

### Electrophoretic profile properties of proteins

The primary and tertiary structure of proteins was examined by SDS-PAGE to estimate molecular weights of polypeptides and their complexes and to compare their respective band intensities.

In 1991 the proposed nomenclature of kafirin polypeptides (subunits) was established based on their similarities with those of zein^22^. More recent studies have revealed greater hydrophobicity with more dominance of α-helical secondary structure in grain kafirin than zein^4,23^. However, zein is still considered an appropriate polypeptide identification standard for kafirins and was used as an internal control in this study.

**Figure 1:**
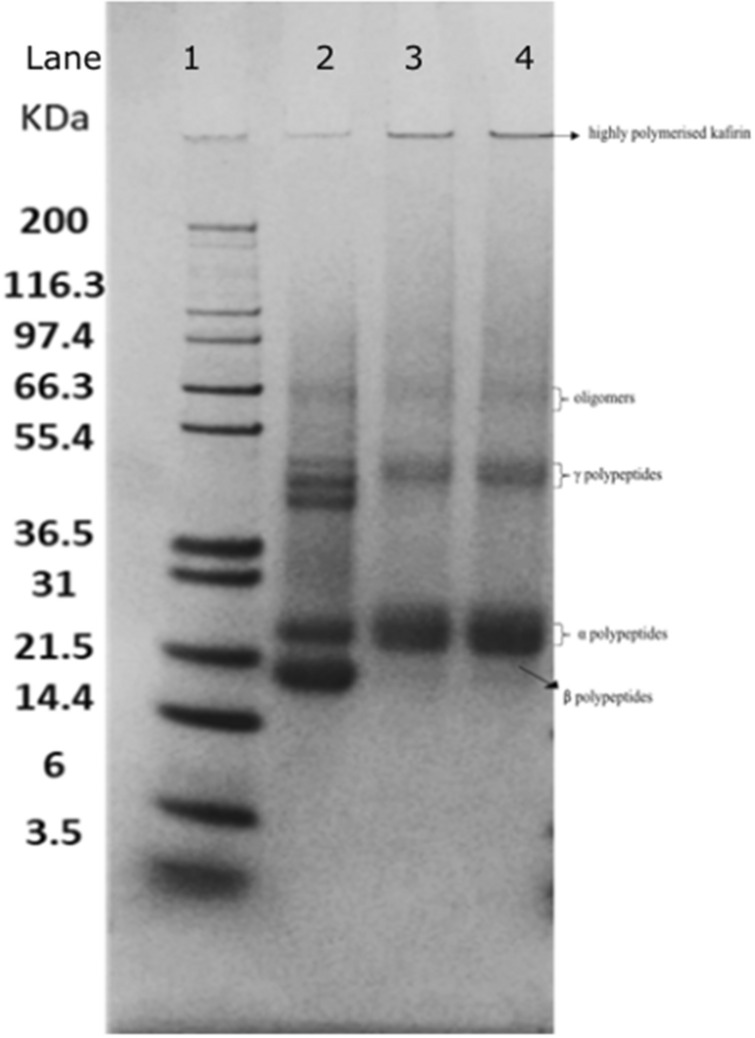
Sodium dodecyl sulphate—polyacrylamide gel electrophoretograms of proteins under non reducing conditions. Lane 1: protein molecular weight marker; Lane 2: zein internal control; Lane 3: sorghum grain kafirin; Lane 4: kafirin from sorghum dried distillers grain with solubles (DDGS). This is an image of a single full gel representative of the experiment.

A typical electrophoretogram under non reduced conditions of protein marker (lane 1), zein (lane 2) and kafirin from sorghum grain (lane 3) and sorghum DDGS kafirin (lane 4) is given in Fig. 1. For zein, the banding pattern was in line with those previously reported^22,24,25^. The zein shows a band at ~ 19 kDa corresponding to β polypeptide monomer, a band at ∼ 21–23 kDa corresponding α_1_ and α_2_ polypeptides. A visible band from ∼ 43 to 50 kDa is indicative of γ polypeptides and a few faint oligomer bands are seen at ∼ 63 to 67 kDa.

Kafirin from both sorghum grain and DDGS had very similar banding patterns and our banding pattern for zein is in line with the previous findings for this protein^26,27^. Both kafirins had a major band at ∼ 21 to 23 kDa indicative of α_1_ and α_2_ kafirin polypeptides^4^. A faint band was seen in both kafirins seen at ∼ 20 kDa indicative of β polypeptide monomer^28^. A band at ∼ 28 kDa is likely to be co-migration between an α polypeptide and a γ polypeptide. The band at ∼ 47 kDa seen in both kafirins corresponds to the γ polypeptide^29^. There is however some ambiguity in the literature about the band at ∼ 50 kDa because it has been reported that it separates into smaller polypeptides under reducing conditions suggesting a possibility of a dimer^7,12^. Faint bands at ∼ 67 to 86 kDa are also seen corresponding to oligomers and a distinct band at the bottom of the loading wells indicating presence of highly polymerized kafirins that were not able to enter the gel.

The similar electrophoretic patterns of both grain and the DDGS kafirins indicate that the kafirin polypeptides were stable to the harsh conditions used in the sorghum bioethanol production process (eg. heat and pressure) that resulted in the DDGS.

### Characterising protein secondary structure using attenuated total reflection-Fourier transform IR spectroscopy (ATR-FTIR)

**Figure 2:**
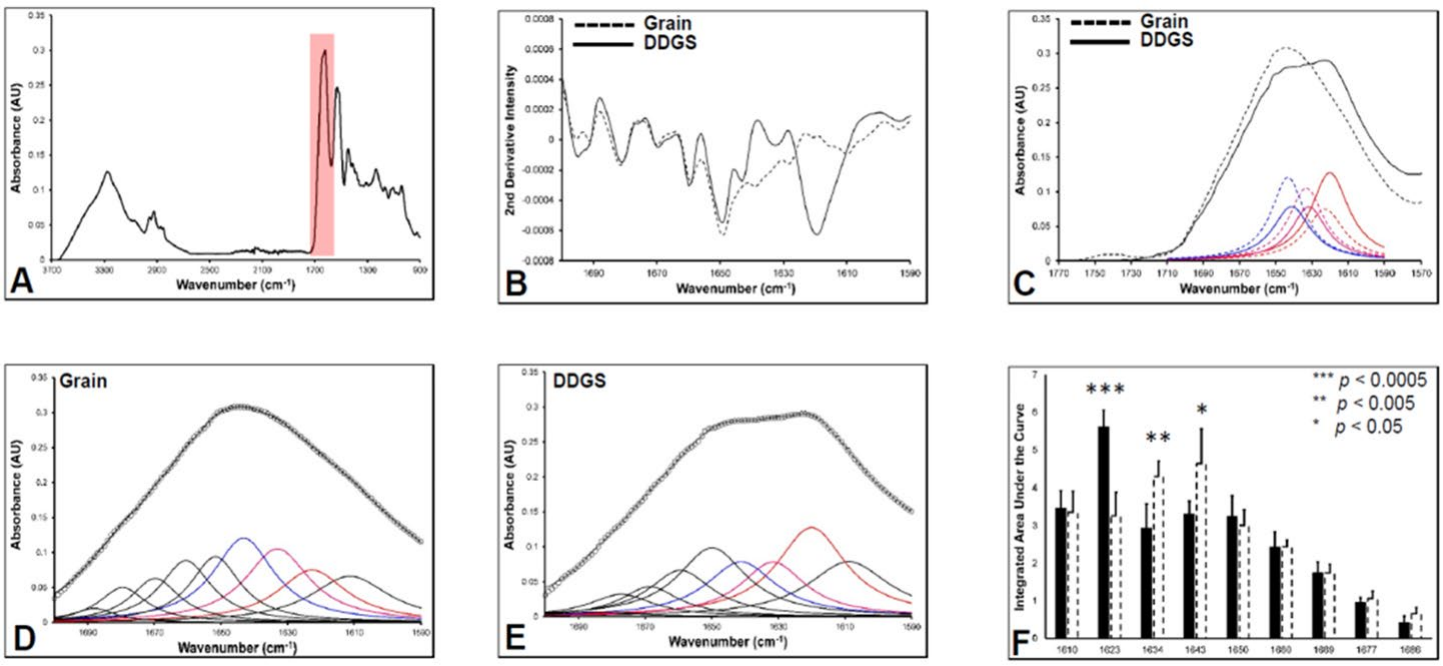
Attenuated total reflection-Fourier transform infra-red (ATR-FTIR) spectroscopics characterisation of protein secondary structure. (**A**) A representative spectra of the kafirin with the amide I band shaded in pink; (**B**) second-derivative spectra; (**C**) the representative overlay of fits centred at 1620, 1634, and 1643 cm^−1^. DDGS kafirin is shown as solid red/pink/blue lines, grain kafirin shown as dashed red/pink/blue traces. (**D**) A representative complete fits for grain kafirin; (**E**) a representative complete fits for DDGS kafirin; (**F**) statistical analysis (n = 5 samples, students’ *t* test, 95% confidence interval) of examined peaks.

Characterising the secondary structure of proteins (e.g., prevalence of β-sheets, α-helices) is important as it can affects their functionality in related to material behaviour. No differences were visible in FTIR spectrum of the two kafirins. Figure 2A is a representative FTIR spectrum the kafirin, showing the characteristic amide I (νC=O of amide functional group) and amide II (δN–H of amide bond) bands. The amide I band is shaded in pink, the amide II band is that directly to the right. It is well established in the literature that that the amide I band is highly sensitive to the molecular geometry and conformational state of the protein secondary structure because (1) different secondary structures are stabilised by the inherently unique patterns of hydrogen-bonding across the carbonyl group in the amide linkage, (2) the νC=O vibrational frequency of the amide group is altered by hydrogen bonding^30–33^. Therefore, the position and shape of the amide I band serves as a fingerprint of protein secondary structure^31,34^. Although absolute determination of protein secondary structures is difficult from analysis of the amide I band alone, identification of relative changes in protein secondary structure has been routinely undertaken by many, including in previous studies of kafirin^31,34–37^.

Although appearing as a single broad peak in the raw data (Fig. 2A), the amide I band is actually the result of multiple overlapping absorbance bands, each representing an underlying secondary structure. The position of the underlying bands can be identified through spectral deconvolution approaches, such as calculation of second-derivatives (Fig. 2B), which artificially decrease band-width enhancing spectral resolution. As can be seen in Fig. 2B, the amide I band of both kafirins consist of 9 underlying bands, centred at ~ 1686, 1677, 1669, 1660, 1650, 1643, 1634, 1623, and 1610 cm^−1^. These second-derivative spectra indicate a difference in secondary structure between grain kafirin and DDGS kafirin by a greater secondary derivative intensity at 1620 cm^−1^ in the latter.

To validate the results of the second-derivative spectra of the kafirins, curve fitting approaches were used to the fit original non-derivatised spectrum to a linear combination of underlying components. Curve fitting was performed using a linear least squares algorithm, and included two additional bands to those identified from second-derivatives, to account for absorbance contribution from the neighbouring ester carbonyl and amide II bands. The relative distribution of underlying components of peaks with major difference as examined on second derivative spectra centred at 1620, 1634, and 1643 cm^−1^ of DDGS kafirin compared to grain kafirin is shown in Fig. 2C. These overlapping fits confirm altered protein secondary structure shape between DDGS kafirin and grain kafirin.

The complete curve fitting to determine the area underneath each of 9 underlying bands of amide I of the second derivatives for the two kafirins are represented in Fig. 2D (grain kafirin) and Fig. 2E (DGGS kafirin). A full assignment of each underlying amide I component to a protein secondary structure is beyond the scope of this paper. However, on comparing curve fits, that for DDGS kafirin appears to have higher contribution for the underlying component centred around 1620 cm^−1^ and lower contribution from underlying components centred at 1643 cm^−1^ and 1634 cm^−1^ than grain kafirin. The integrated areas under the curves of amide I as determined by curve fitting Fig. 2D, E were then statistically analysed (n = 5, students’ *t* test, 95% confidence interval) and presented in Fig. 2F. The key finding this study supports increase in area of peak centered at 1620 cm^−1^ (***p < 0.0005) but also suggest decrease in area of peak at 1643 cm^−1^ (*p < 0.05) and 1634 cm^−1^ (**p < 0.005). It has been established extensively in the literature^38^ that the underlying component at ~ 1623 cm^−1^ likely results from proteins with an aggregated β-sheet secondary structure.

The band centred around 1643 cm^−1^ is likely to arise from random or disordered secondary structures, and the band at 1634 cm^−1^ is likely to arise from non-aggregated β-sheets. There is some ambiguity in these assignments however, as the band at 1643 cm^−1^ could also contain contributions from 3_10_Helices, or even from strongly hydrogen bonded α-helices. Nonetheless, regardless of this ambiguity, the results strongly support a higher level of extended β-sheet aggregates and lower levels of α-helix and random coils in DDGS kafirin.

Such findings are consistent with past literature were wet cooked sorghum and maize proteins were analysed using FTIR^39^, showing that the amide I absorbance peak centred at ~ 1635 cm^−1^ increased in intensity on cooking. The authors revealed this increase in intensity is a result of breakdown of hydrogen bonds by energy generated upon wet cooking. Also, this breakdown resulted in a decreased peak area centered at ~ 1643 cm^−1^ as this peak represents the α-helix is mainly stabilised by intramolecular hydrogen bonds.

Also, our spectra agree with those seen after high temperature treatment of purified kafirin, which also indicates a propensity of the protein to produce aggregated β-sheet structures at high temperatures^36^. Similarly, Ezeogu et al.^40^ reported secondary structural changes in prolamin proteins from sorghum and maize on cooking e.g., a shift in band intensities of amides. The authors presumed that during cooking some intra and intermolecular disulfide bond breakdown had led to these structural changes.

We hypothesise that this is because of during the biofuel manufacturing process the high temperature were capable enough to unravel some α-helices and random coils followed by realignment and reorganisation into β-sheets. The higher level of β-sheets in DDGS kafirin compared to grain kafirin may make the former better suited for viscoelastic self-assembly delivery systems. It is well documented in literature that biomaterial with viscoelastic properties have high energy absorption capacity, shock absorbance behaviour and dumping response^41–43^. Thus, it become evident that DDGS kafirin might retain architecture of formulated biomaterial system in physiological environment than that of commonly used elastic biomaterials which are usually thought to be loading dependent only. The DGGS kafirin may endow interfacial wettability to biomaterials because of more aggregated β-sheets, which are believed to be less hydrophobic than α-helix.

### Characterising protein diffraction patterns using X-ray diffraction (XRD)

**Figure 3:**
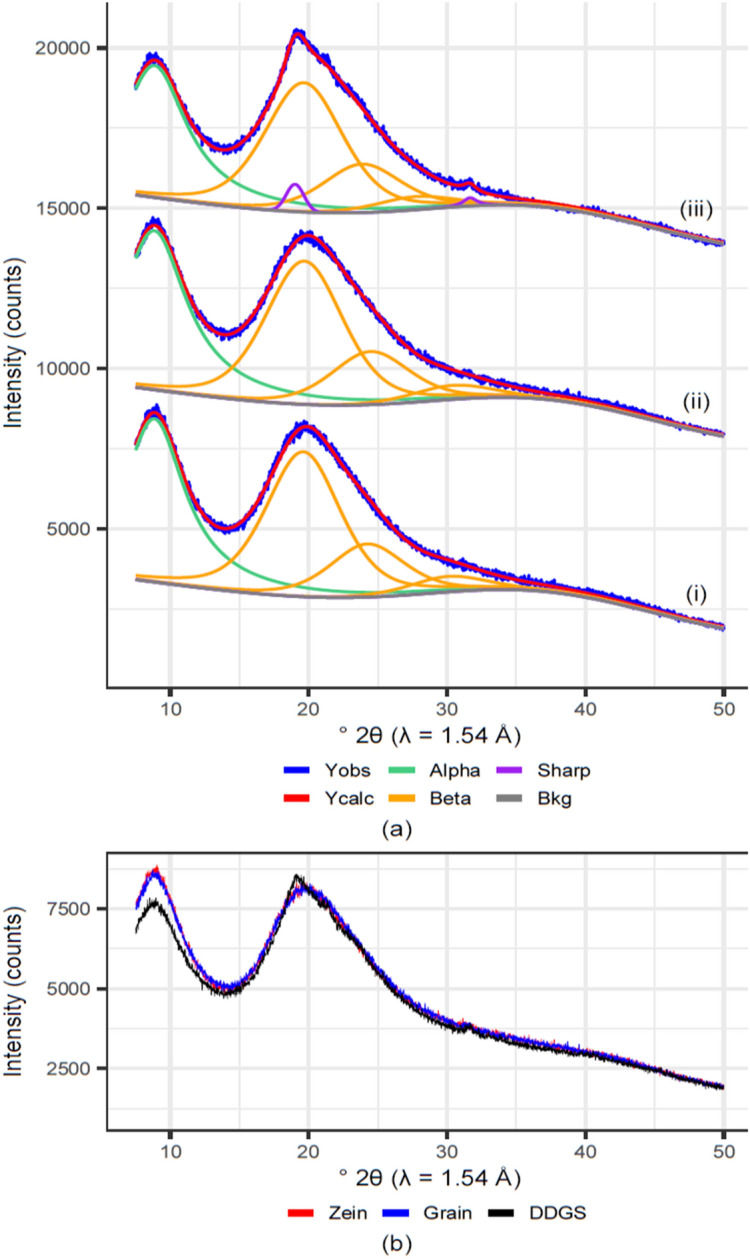
X-ray diffraction patterns, showing fits for Zein (**a-i**), grain kafirin (**a-ii**), and kafirin from dried distillers grain with solubles (DDGS) (**a-iii**). (**b**) The increased intensity at 20° 2θ, and increased 20° to 9° 2θ area ratio in DDGS kafirin compared to grain kafirin and zein.

The XRD patterns of DDGS kafirin, grain kafirin and zein exhibit two broad peaks at approximately 9° and 20° 2θ (Fig. 3a), which is consistent with previous XRD investigations of prolamin proteins^20,44^. In general, diffraction studies are not “information rich” with respect to determining the structure of prolamins, largely due to a high degree of unordered structure within prolamins, which may be amorphous or nano-crystalline^45,46^. The results of this study are consistent with the literature and indicate a low degree of order of nano-crystalline structure in the zein, grain kafirin and DDGS kafirin, which is evident through the large widths of the peaks centred at ~ 9° and 20° 2θ. As the peak centred at 20° 2θ was asymmetric, curve fitting was undertaken. The results indicate that 3 underlying curves (at ~ 19.5°, 24°, and 30° 2θ, all constrained to have the same peak width) are required to fit the peak at 20° 2θ.

The Fig. 3b, highlights increased intensity at 20° 2θ, and increased 20° to 9° 2θ area ratio in DDGS kafirin. The impact of this final peak on the XRD patterns can be observed as an altered, more narrow and intense peak shape at 20° 2θ in the pattern of kafirin DDGS. Likewise, the ratio of the peak areas of the peak at ~ 9° 2θ and 20° 2θ is slightly lower in DDGS kafirin than the zein or grain kafirins.

These differences in the diffraction patterns in DDGS kafirin relative to zein and grain kafirin support a difference in protein structure, most likely reorientation^47^ and packing of the molecules in to a more ordered arrangement^48^ in the DGGS kafirin. As the FTIR spectroscopic data (Section "Characterising protein secondary structure using attenuated total reflection‑Fourier transform IR spectroscopy (ATR‑FTIR)") indicated increased β-sheet aggregates in the DDGS kafirin, which are often fibrillar in nature, an increased in β-sheet fibrils in DDGS kafirin could account for these XRD results.

Another important observation from the XRD data is that although differences in structure between grain kafirin and DDGS kafirin and are apparent, the crystallite size does not appear to have changed substantially (Table 1).

**Table 1:** Diffraction peak position, crystallite size and relative peak areas for zein, grain kafirin and dried distillers grain with solubles (DGGS) kafirin as measured by cx-ray diffraction (XRD).

Sample	Phase	Peak position (° 2θ)	Crystallite size, Lvol (nm)	Area (net)	Area (rel.)
Zein	Alpha	8.87	1.1	128	0.123
	Beta	19.6	1.1	565	1
		24.2		324	
		30.0		152	
Grain kafirin	Alpha	8.87	1.0	132	0.127
	Beta	19.7	1.1	565	1
		24.4		332	
		30.4		141	
DDGS kafirin	Alpha	8.9	1.0	109	0.118
	Beta	19.6	1.1	517	1
		23.8		292	
		27.7		110	
	Sharp	19.0	5.6	19.6	
		31.6	5.0	19.7	

### Characterising protein in-solution structure using small-angle X-ray scattering (SAXS)

**Figure 4:**
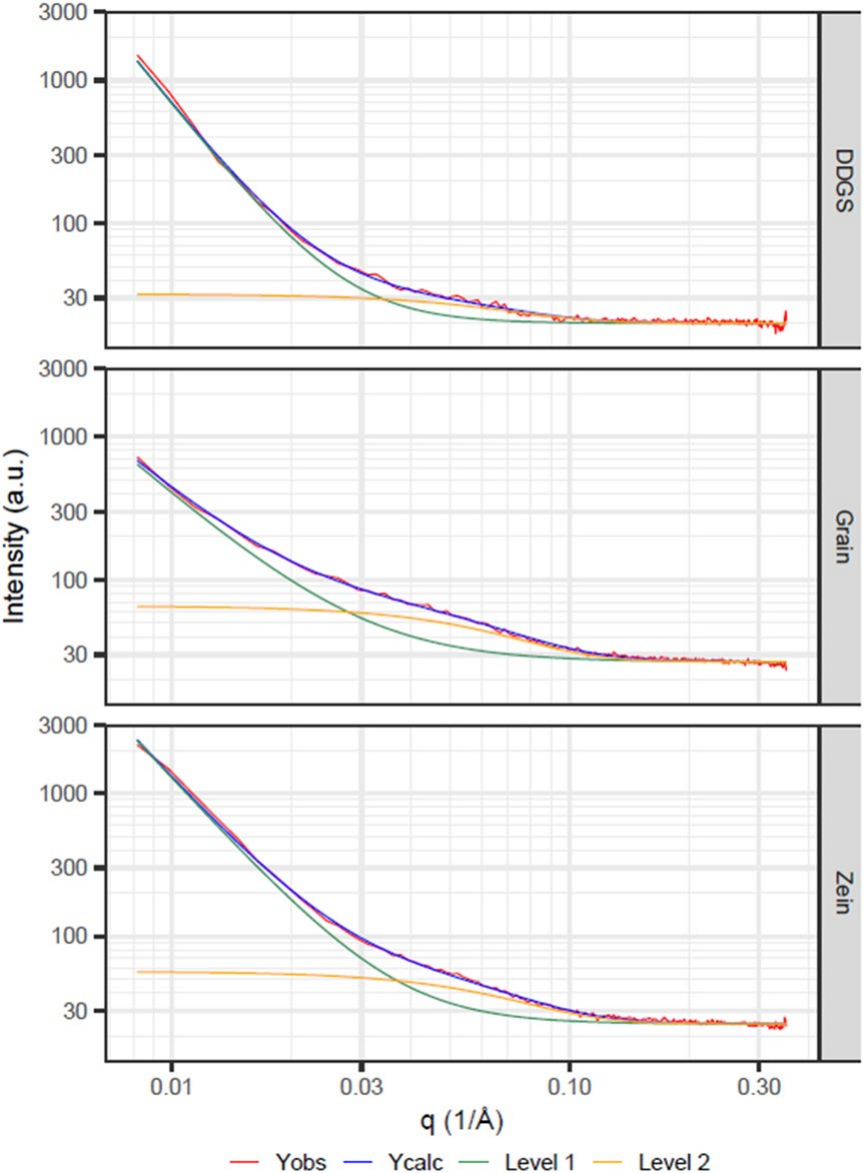
Small angle X-ray scattering (SAXS) patterns, showing the overall model and two individual unified-fit levels for zein, grain kafirin, and kafirin from sorghum dried distillers grain with solubles (DDGS).

SAXS was used to assess morphological features of zein and the kafirins. The scattering curves were well-fitted with a two-level unified fit model^19^ combining Porod and Gunier models, where the first level describes surface scattering from very large particles, and the second level describes scattering from ~ 25 Å particles (Fig. 4).

At low scattering angles, the data can be modelled with a Porod exponent of ~ 3, ~ 2.4 and ~ 3.5 for zein, grain kafirin and DDGS kafirin, respectively^49^. These exponents show that the proteins show properties of surface (3 < P < 4) and volume (P < 3) fractals, showing self-similar behaviour in their structure. If there is no interatomic interaction between molecules, radius of gyration (*R*_*g*_) can be directly linked with mean square of interatomic distances of molecules present in solution medium^50^, allowing the average size of zein and kafirins to be determined. The calculated radii of gyration were 25.8, 24.8, and 28.4 Å for zein, grain kafirin and DDGS kafirin, respectively. These data indicate a difference in the tertiary/quaternary structure of DDGS kafirin, as indicated by the increased radius of gyration, relative to zein and grain kafirin.

### Characterising protein surface morphology and elemental composition using field emission-scanning electron microscopy (FE-SEM) and energy-dispersive X-ray spectroscopy (EDS)

**Figure 5:**
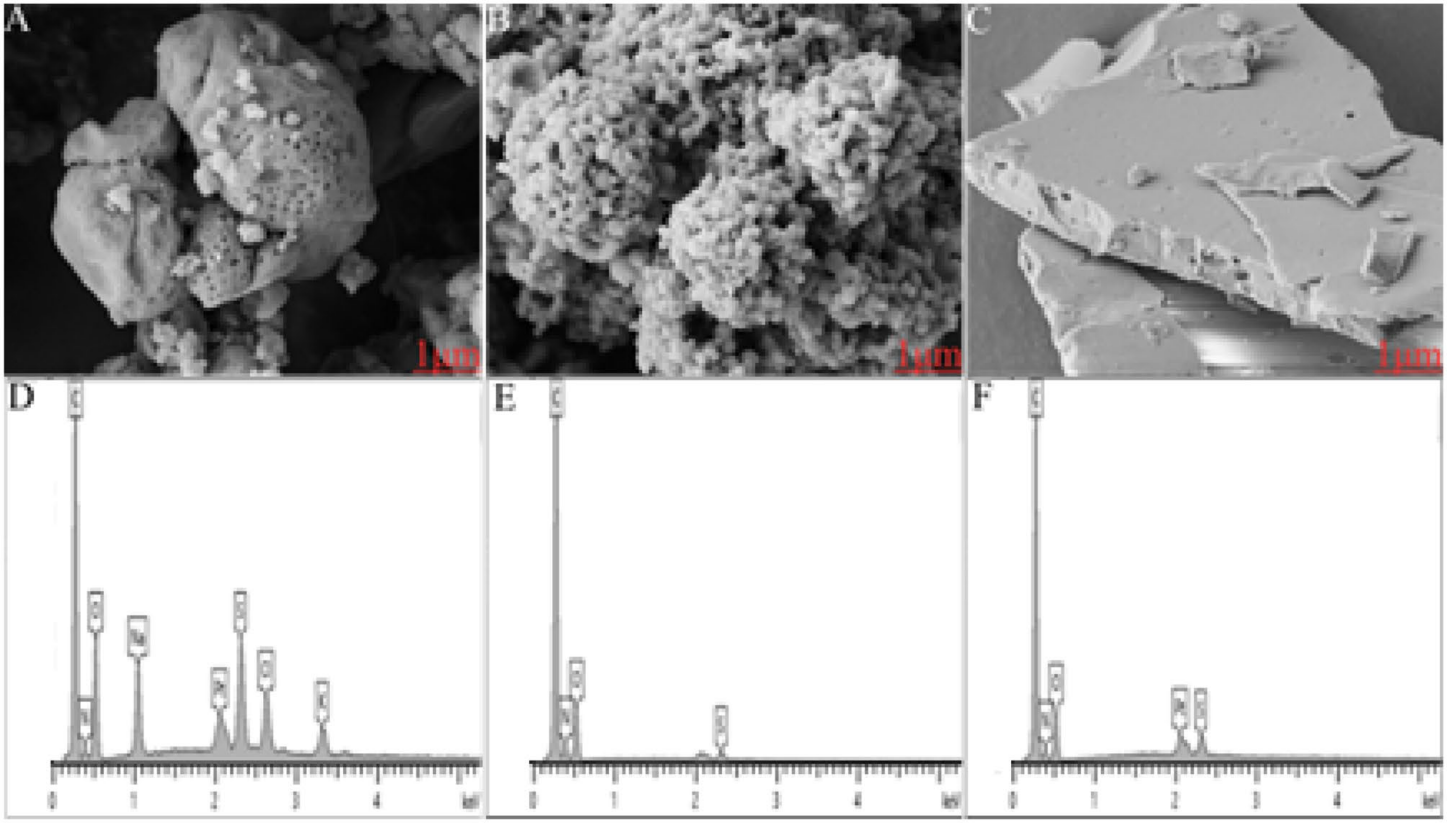
Field emission-scanning electron micrograms (FE-SEM) of proteins at magnification of 1 μm, voltage of 5.00 V, aperture size of 20.00 μm using secondary electron beam signal. (**A**) Kafirin from dried distillers sorghum grain with solubles, DDGS; (**B**) grain kafirin; (**C**) zein. Energy dispersive X-ray spectroscopy of proteins. (**D**) DDGS kafirin; (**E**) grain kafirin (**F**) zein.

The morphological analysis and surface chemical composition of the proteins were examined by FE-SEM and EDS. Figure 5A–C depicts morphological micrographs and Fig. 5D–F the surface elemental composition of DDGS kafirin, grain kafirin and zein respectively.

The DDGS kafirin shows irregular surfaced large aggregates of shapes with internal pores, Fig. 5A while grain kafirin was more spherical with homogeneous surface, Fig. 5B and zein showed non uniform surface assembling characterises, Fig. 5C. The formation of compact micro spherical shapes with more homogeneity in grain kafirin. Thus, it can be hypothesis that assembling grain kafirin into ordered structure need no external guidance but relies on internal interactions such as vander walls, hydrogen bonding (S–S), capillary and π–π. However solvent in use and size of molecule (η/μ) might be of interest in formation mechanism.

The formation of the larger particles in DDGS kafirin might be related to heat induced transformations in the higher structures during manufacturing of ethanol. This is in agreement with the FTIR data which shows higher abundance of aggregated β-sheets in kafirin DDGS. Heat induced disruption of disulphide bonds (S–S) resulting in re-association of polypeptides into large compact micro aggregates^51–53^ could be a possible pathway to aggregated β-sheet formation. Another possible formation mechanism for the aggregates is that some polypeptides were denatured during the bioethanol production system leading to a subsequent increased propensity to form aggregates.

The elemental profile across the surface of DDGS kafirin, grain kafirin and zein was examined by EDS, and is represented in Fig. 5D–F. The EDS showed an abundance of C, N, O and S on the surface of grain kafirin Fig. 5F and zein Fig. 5E. The zein literature suggested formation of surface segments with these elements have high hydrophobicity with contact angle of 126°^21^. The presence of Pt in DDGS kafirin and zein is because of the sputter coating.

The surface elemental composition of DDGS kafirin was different than grain kafirin and zein. In addition to C, N, O and S elements some more elements Na, Cl and K were present, Fig. 5D. It is a well-known fact that elements facing bottom left corner of periodic table such as sodium, potassium and etc. are more active suggesting increased reactive surface sites for DDGS than grain kafirin and zein.

The novelty of the DDGS kafirin is that despite presence of the additional elements on surface, atomic radius of C and N element on surface remained same and atomic radius of S and O element increased. Although, additional elemental profile gives a clear understanding that DDGS kafirin might endow interfacial wettability. Thought striking, increased atomic radius of elements (S & O), which are proven to form hydrophobic surface sites^21^, and additional more reactive elements suggest DDGS surface might have both hydrophobic and hydrophilic surface segments.

Thus, we hypothesise that compared to grain kafirin, DDGS kafirin if used as an active encapsulating agent might have enhanced solubility and formation of complexes with target compounds in aqueous systems. These differences in structure and functionality may also be explained by heat induced transformations in the molecular architecture of DDGS kafirin during the bioethanol production process.

## Conclusion

A detailed comparison of physicochemical characteristics of sorghum, grain kafirin and sorghum DDGS kafirin identified differences between the two types that likely arose from heat induced molecular changes to the DGGS kafirin during the bioethanol production process. The primary and quaternary structures of the two kafirins appeared similar through electrophoretic examination. However FTIR results indicated that the DDGS kafirin had higher levels of extended β-sheet aggregates which in turn gave higher protein order indicated by XRD. These properties of DGGS kafirin may affect dissolution behaviour which in turn greatly affects other techno-functionalities such as gelation properties including foaming stability, emulsifying properties, swelling and solubility index required for biomaterial fabrication.

The in-solution morphological structure indicate DDGS kafirin with surface fractal. However, this study suggested that only minimal processing should be required for assembling of DDGS kafirin based biomaterials because of (1) low and self-similar Porod exponent to that of grain kafirin, which has self-assembling capacity (2) same crystalline size to that of grain kafirin as observed by XRD.

Morphological chemical examination also supported that DDGS kafirin might be better suited for material behaviour because (1) it contains both hydrophobic and hydrophilic surface segments, which are thought to ease loading capacity (2) additional elements might endow interfacial wettability (3) easy chemical reactivity with the assigned molecule, atom or a compound eg during encapsulation.

These behaviour properties investigated in this study should act as fundamental information to better understand DDGS kafirin functionality and upgrade it for biomaterial material use. Future studies should determine the potential of DGGS kafirin as cost effective raw material for biomaterials. For example investigating its efficacy in applications such as microparticle fabrication for encapsulation and controlled delivery of bioactives and drugs for nutraceuticals and pharmaceuticals.
